# Cost-effectiveness analysis of robotic-arm assisted total knee arthroplasty

**DOI:** 10.1371/journal.pone.0277980

**Published:** 2022-11-28

**Authors:** Yechu Hua, Jonathan Salcedo

**Affiliations:** 1 Department of Public Health Sciences, Division of Health Policy and Outcomes Research, University of Rochester Medical Center, Rochester, NY, United States of America; 2 Analysis Group, Inc., Boston, MA, United States of America; Taipei Medical University, TAIWAN

## Abstract

**Purpose:**

Total knee arthroplasty (TKA) is widely recognized as an effective treatment for end-stage knee osteoarthritis (OA). Compared with conventional TKA, robotic-arm assisted TKA may improve patients’ functionality and resulting quality of life by more accurate and precise component placement. Currently, the literature on cost-effectiveness of robotic-arm assisted TKA in the US is limited. The objective of this study was to assess the cost-effectiveness of robotic-arm assisted TKA relative to TKA in the Medicare-aged population including exploring the impact of hospital volume on cost-effectiveness outcomes.

**Methods:**

We developed a decision-analytic model to evaluate the costs, health outcomes, and incremental cost-effectiveness ratio (ICER) of robotic-arm assisted TKA vs TKA in Medicare population with OA. We evaluated cost-effectiveness at a willingness-to-pay (WTP) threshold of $50,000 per quality-adjusted life-year (QALY). We sourced costs from the literature including episode-of-care (EOC) costs from a Medicare study. We assessed cost-effectiveness of robotic-arm assisted TKA by hospital procedure volume and conducted deterministic (DSA) and probabilistic sensitivity analysis (PSA).

**Results:**

For the average patient treated in a hospital with an annual volume of 50 procedures, robotic-arm assisted TKA resulted in a total QALY of 6.18 relative to 6.17 under conventional TKA. Total discounted costs per patient were $32,535 and $31,917 for robotic-arm assisted TKA and conventional TKA, respectively. Robotic-arm assisted TKA was cost-effective in the base case with an ICER of $41,331/QALY. In univariate DSA, cost-effectiveness outcomes were most sensitive to the annual hospital procedure volume. Robotic-arm assisted TKA was cost-effective at a WTP of $50,000/QALY only when hospital volume exceeded 49 procedures per year. In PSA, robotic-arm assisted TKA was cost-effective at a $50,000/QALY WTP threshold in 50.4% of 10,000 simulations.

**Conclusions:**

Despite high robotic purchase costs, robotic-arm assisted TKA is likely to be cost-effective relative to TKA in the Medicare population with knee OA in high-volume hospitals through lowering revision rates and decreasing post-acute care costs. Higher-volume hospitals may deliver higher value in performing in robotic-arm assisted TKA.

## Background

Total knee arthroplasty (TKA) is widely recognized as an effective treatment for end-stage knee osteoarthritis (OA). In the United States (US), TKA is one of the most expensive surgical procedures among adult patients, with average total costs ranging from $31,558 to $37,370 [1–4]. Demand for primary TKA is projected to increase substantially due to the aging of the population and increased prevalence of obesity, with a projected annual volume of over a million by 2030 [5–7]. Surgical complications after TKA are common and costly. One published systematic review and meta-analysis found the overall complication rate was 3.3% at 30 days and 9.7% at 90 days [8]. These complications had an average cost $38,953 (range: $4,790-$104,794) for TKA [9].

Robotic-arm assisted TKA is an alternative to TKA which has the potential of reducing costly complications and readmissions with robot-assisted techniques. Before surgery, a CT scan is utilized to produce a 3D model of the patient’s knee, allowing doctors to place the implant more precisely [10, 11]. During the procedure, the robotic arm uses data from the 3D model to establish a specified working space for the surgeon, preventing them from injuring the surrounding tissue [12, 13]. Though long-term outcomes of robotic-arm assisted TKA are not available, preliminary clinical studies showed that robotic-arm assisted may improve functional outcomes and patient satisfaction [14–17]. In addition to the potential improvement in outcomes, an economic analysis found that robotic-arm assisted was associated with significantly lower 90-day episode-of-care (EOC) costs [18]. The studies on clinical outcomes and costs suggested that robotic-arm assisted has the potential of reducing costly complications and readmissions with robot-assisted techniques.

The literature on cost-effectiveness of robotic-arm assisted vs conventional TKA in the US is limited. Following a review of the literature, we identified two published studies comparing the cost-effectiveness of these two interventions [19]. Using a Markov decision model, the study by Vermue et al. provided preliminary cost-effectiveness estimates of robotic-arm assisted TKA and their results suggested robotic-arm assisted TKA might be a cost-effective procedure if annual surgical volume exceeded 253; Rajan et al. also used a Markov decision model and found that robotic-arm assisted TKA might be cost-effective when case volume > 24 cases per year [20]. However, neither of the studies consider significant cost savings of robotic-arm assisted TKA in the post-discharge phase. This study aims to expand upon the literature on cost-effectiveness of robotic-arm assisted TKA in the Medicare-aged population with knee OA including exploring the impact of hospital volume on cost-effectiveness outcomes.

## Model overview

Given robotic-arm assisted TKA’s potential to improve patient outcomes and reduce costs, particularly in higher procedure volume hospitals, we used a decision-analytic model to assess the cost-effectiveness of robotic-arm assisted vs conventional TKA using Microsoft Excel. The model parameters were obtained from published studies on transitional probabilities, costs, and outcomes of both conventional and robotic-arm assisted TKA. Our study doesn’t involve human subjects or personal level data.

### Decision model

**Fig 1:**
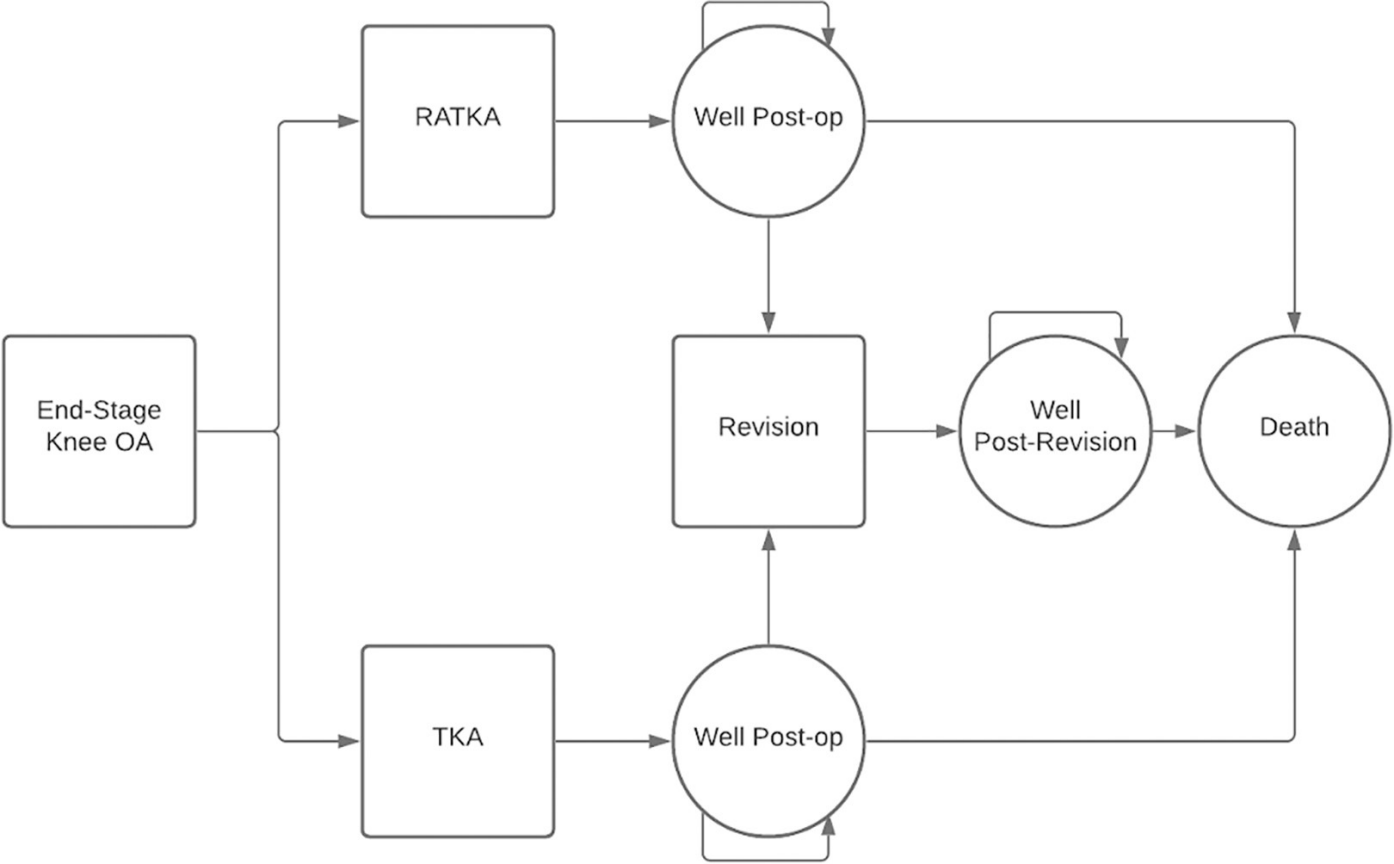
The decision-analytic model. **Abbreviations**: OA: osteoarthritis; RATKA: robotic-arm assisted total knee arthroplasty.

We developed a decision-analytic model composed of decision node and a recurring Markov process to evaluate a cohort of Medicare beneficiaries aged 65 years undergoing treatment for end-stage knee OA. In our base case, we assumed patients received care from a hospital with an annual volume of 50 procedures. Fig 1 depicts the model used to characterize a sequence of transitions among health states following primary TKA. We used the model to examine the incremental clinical improvement and costs of robot-assisted vs traditional guides for patient undergoing primary. Each patient undergoes primary TKA with or without robotic assistance. After a year spent in the TKA state, patients transition to the “well postoperative state” and are at an annual risk of TKA failure. If a failure occurs, patients transition to the revision TKA state, and their health-related quality of life (HRQoL) will be worse than the pre-revision state. The model only allows for one revision TKA over a horizon of 10 years, which is consistent with general clinical evidence [21]. Patients are always at risk of death from acute states (robotic-arm assisted TKA, TKA, and revision) and chronic states. We calculate the present value of direct medical costs (in 2021 US dollars) and QALYs both using a 3% annual discount rate [22]. The cycle length was yearly beyond year 1.

### Populations under consideration

TKA is a safe and common inpatient procedure that relieves pain and improves the functional status of patients with advanced knee osteoarthritis, the most common type of arthritis among older people [23]. Therefore, we modeled a Medicare population (65 years or older) with end-stage knee OA.

### Model assumptions

We made several assumptions in the decision-analytic model. First, robotic arm-assisted TKA was first introduced in 2006 thus long-term patient outcomes are not yet available in published studies [24]. Therefore, QALY weights following the initial TKA were assumed to be the same regardless of the use of robot assistance. Second, perioperative mortality rates were the same for both robotic-arm assisted TKA and conventional TKA. Third, revision TKA was performed without robotic assistance regardless of the initial surgery. Fourth, the costs of the robotic system were the same across hospital volume groups.

### Model parameters

#### Transitional probability

*Mortality rate*. Based on a nationwide study on Medicare beneficiaries, we used 90-day perioperative mortality rates of 1.1% and 0.7% for primary TKA and revision TKA respectively [25]. Because no published studies report data on the perioperative mortality rate of Robotic-arm assisted TKA, we assume his parameter to be the same for both robotic-arm assisted TKA and conventional TKA. For the revision TKA, we assume that all patients undergo the conventional procedure regardless of their primary surgery. Overall age-specific mortality rates were taken from general US population life tables by the Centers for Disease Control and Prevention [26].

*Revision rate*. Over time, failure of the initial TKA might occur and require revision surgery. Using estimates from a 10-year follow-up study on 71 robotic-arm assisted TKAs, we set the annual probability of revision at 0.28% for robotic-arm assisted TKA [27]. Using the estimate from a systematic review on 124 studies, we set the annual revision rate of conventional TKA at 0.49% [28].

#### Health utilities

Health utilities in the model were defined as quality-adjusted life-year (QALY) weights and were identified from a review of the literature. HRQoL after undergoing TKA with or without robotic assistance was set using a QALY weight of 0.835 [29]. Similar to Moschetti et al, quality of life following revision surgery was set using a QALY weight of 0.565.

*Disutilities*. In the model, we assessed disutilities when a patient undergoes either primary or revision surgery. The disutilies represent the diminished HRQoL during the perioperative period. The disutility of the initial surgery was omitted in the analysis as both robotic-arm assisted TKA and conventional TKA groups would experience the same magnitude of disutility. Similar to Slover et al., the disutility of having revision surgery was set at 0.1 which is equivalent to losing one-tenth of a year of perfect health [30].

#### Costs

We considered 90-day episode-of-care (EOC) costs from a healthcare sector perspective, including intraoperative, inpatient, postoperative costs. For the robotic system and associated costs, we obtained them from Cotter et al. [18] who sourced these numbers from an academic institution in Wisconsin that performed around 600 robotic cases annually. The system was expected to have a 10-year lifetime. We depreciated the initial purchase costs of $1.3 million over this 10-year period. Fixed costs, including annual maintenance fees, were summed and divided over annual TKA cases plus disposable costs per case. Other costs that occurred during the episode of care were estimated using Medicare Inpatient Prospective Payment data. Notably, robotic-arm assisted TKA generated cost savings in both inpatient and postoperative phases from shorter length of stay and reduced postoperative resource utilization [31]. All the costs were converted to 2021 US dollars using the medical component of the Consumer Price Index (CPI).

#### Analysis

Primary outcomes of our analysis were total QALYs, total costs, the incremental cost-effectiveness ratio (ICER), and incremental net monetary benefits (INMB) of robotic-arm assisted TKA relative to TKA. We considered 3 commonly used willingness-to-pay (WTP) thresholds: $50,000 per QALY, $100,000 per QALY, and $150,000 per QALY [32]. Robotic-arm assisted TKA was considered to be cost-effective when the ICER was below a specified WTP threshold and INMB was positive.

We used probabilistic sensitivity analysis (PSA) to test the robustness of the analysis by assuming a probability distribution for each parameter and changing all parameters in the model simultaneously. Then we carried out deterministic sensitivity analysis (DSA) where we changed each of the parameters used in the model while holding other parameters constant to identify factors with the greatest impact on cost-effectiveness. The parameters used in sensitivity analysis included perioperative mortality rates, revision rates, QALYs, and specific components of 90-day EOC costs.

## Results

### Base case

Table 1 showed model inputs in the baseline scenario where a hospital performed 50 procedures per year. In a patient population with an average of 65 years old, robotic-arm assisted TKA resulted in slighter higher accumulated costs and a higher number of QALYs in the base case. The average cost difference was only $747.35 while the average improvement in QALYs was only 0.01 over a horizon of 10 years (Table 2). The incremental cost-effectiveness ratio (ICER) of robotic-arm assisted TKA was $41,331 per QALY, below the $50,000 WTP threshold. At the same threshold, the incremental net monetary benefit (INMB) was $129. INMB was calculated as incremental QALYs × WTP − incremental costs. A positive INMB indicates that the robotic-arm assisted TKA is cost-effective compared with conventional TKA at the given willingness-to-pay threshold. Therefore, robotic-arm assisted TKA was cost-effective at a hospital performing 50 cases of robotic arm-assisted TKA.

**Table 1:** Model input parameters.

Parameter	Value	Confidence Interval^4^	Distribution^4^	Source
Transitional Probabilities				
Overall population of death	Age-based table	-		[26]
Perioperative death after TKA	1.10%	[0.09%, 1.3%]	Beta	[25]
Perioperative death after revision to TKA	0.70%	[0.60%, 0.90%]	Beta	[25]
Annual probability of revision to TKA	0.49%	[0.41%, 0.58%]	Uniform	[27]
Annual probability of revision to robotic-arm assisted TKA	0.28%	[0.03%, 0.77%]	Beta	[28]
Quality-adjusted life-years				
Primary TKA	0.835	[0.832, 0.837]	Beta	[29]
Revision to TKA	0.565	[0.45, 0.68]	Uniform	[30]
Disutility of revision to TKA	0.1	[0.08, 0.12]	Uniform	[30]
Costs (2021 USD)^1^				
90-day EOC costs: robotic-arm assisted TKA	$32,018		Gamma	
Intraoperative costs	$13,180		Gamma	[18, 31]
Annual depreciation of robotic system^2^	$2,696	[$907, $6,403]	Gamma	[18]
Maintenance fees	$438	[$147, $1,040]	Gamma	[18]
Disposable cost	$730	[$246, $1,734]	Gamma	[18]
Other intraoperative costs^3^	$10,662	[$6,988, $18,284]	Gamma	[31]
Inpatient costs	$15,937	[$11709, $25384]	Gamma	[31]
Postdischarge costs	$1,618	[$7, $8,212]	Gamma	[31]
90-day EOC costs: TKA	$31,134		Gamma	
Intraoperative costs	$11,231	[$7,765, $18,620]	Gamma	[31]
Inpatient costs	$17,506	[$12,815, $27,952]	Gamma	[31]
Postdischarge costs	$2,397	[$6, $12,670]	Gamma	[31]
Revision to TKA costs	$24,446	[$9,221, $76,094]	Gamma	[2]

**Abbreviations**: EOC: episode of care; TKA: total knee arthroplasty.

Note

1. Cost estimates presented are per case where a hospital performs 50 robotic-arm assisted TKAs annually.

2. The system was expected to have a 10-year lifetime. We depreciated the initial purchase costs of $1.3 million over this 10-year period.

3. Other intraoperative costs include costs associated with preoperative holding area, anesthesia, operating room (OR) supplies, instrument reprocessing, OR time, and implant.

4. The selected distribution and confidence interval for parameters varied in the sensitivity analysis

**Table 2:** Results for base case analysis.

Strategy	Cost	QALY	Incremental Cost	Incremental QALY	ICER	INMB
Conventional TKA	$31,917	6.17	-	-	-	-
Robotic Arm Assisted TKA	$32,535	6.18	$747.35	0.01	$41,331	$129

**Abbreviations**: QALY, quality-adjusted life-year; ICER: incremental cost-effectiveness ratio; INMB: incremental monetary net benefit.

**Note**: The INMB willingness-to-pay (WTP) threshold was assumed to be $50,000 per QALY. INMB was calculated as incremental QALYs × WTP − incremental costs.

### Sensitivity analysis

**Fig 2:**
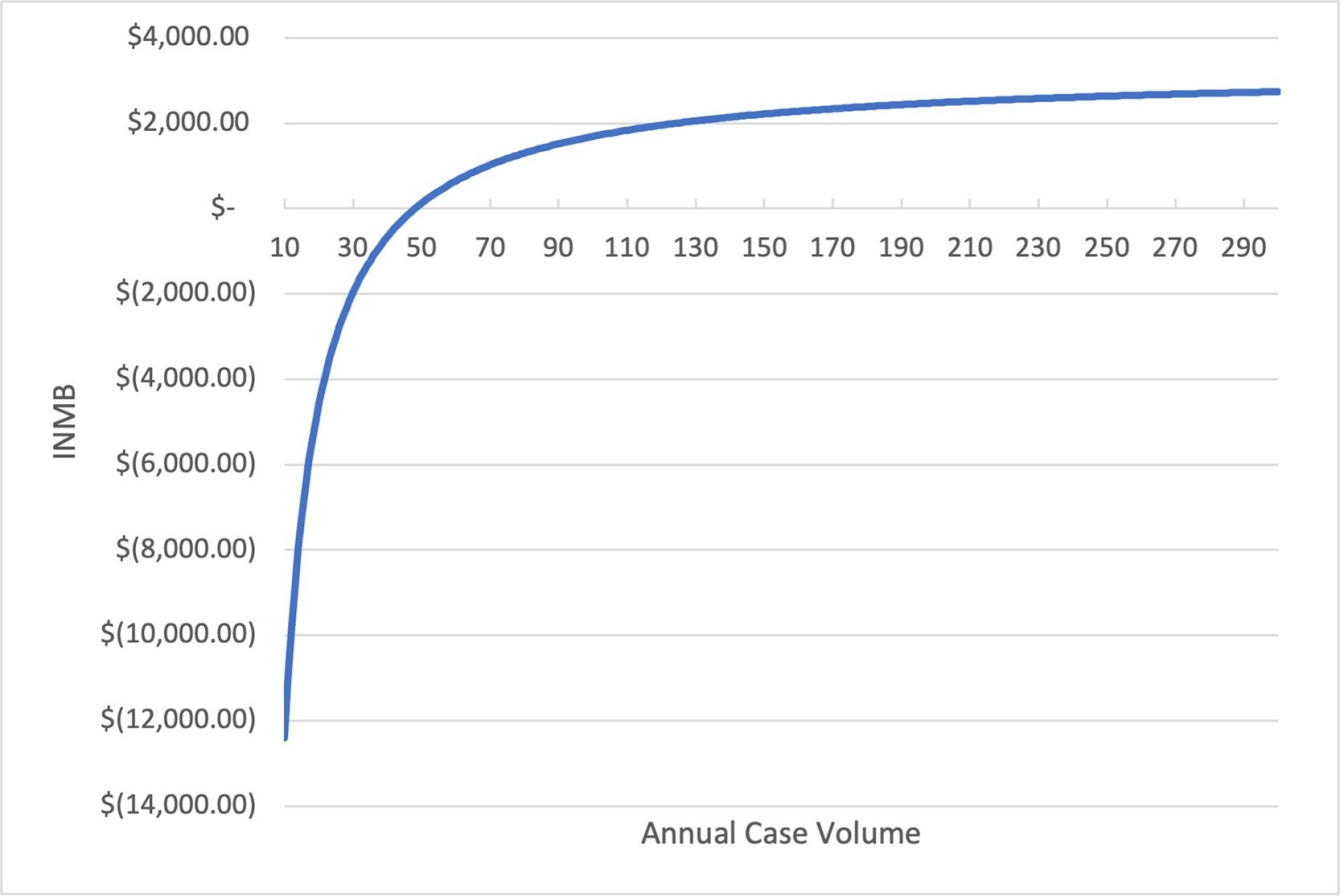
Annual case volume and INMB of robotic-arm assisted TKA. **Abbreviations**: INMB: incremental monetary net benefit; TKA: total knee arthroplasty. **Note**: The INMB willingness-to-pay (WTP) threshold was assumed to be $50,000 per QALY. INMB was calculated as incremental QALYs × WTP − incremental costs. Robotic-arm assisted TKA was cost-effective when case volume exceeded 49 cases per year.

There was an inverse relationship between annual surgical volume and INMB for robotic-arm assisted TKA. Robotic-arm assisted TKA was cost-effective when case volume exceeded 49 cases per year (Fig 2). At a lower-volume hospital, robotic-arm assisted TKA cost significantly more than the conventional TKA. For a hospital that performed only 20 procedures per year, the average accumulated costs of robotic-arm assisted TKA were significantly higher than the conventional TKA (robotic-arm assisted TKA: $37,235 vs TKA: $31,917, difference: $5,318).

The results from probabilistic sensitivity analysis suggested that for a hospital with an annual surgical volume of 50, robotic-arm assisted TKA had a 50.04% chance of being the more cost-effective procedure compared to the conventional TKA at the WTP threshold of $50,000 per QALY gained. If the WTP threshold was set to $100,000 per QALY gained, robotic-arm assisted TKA had a 50.91% chance of being the preferred choice.

**Fig 3:**
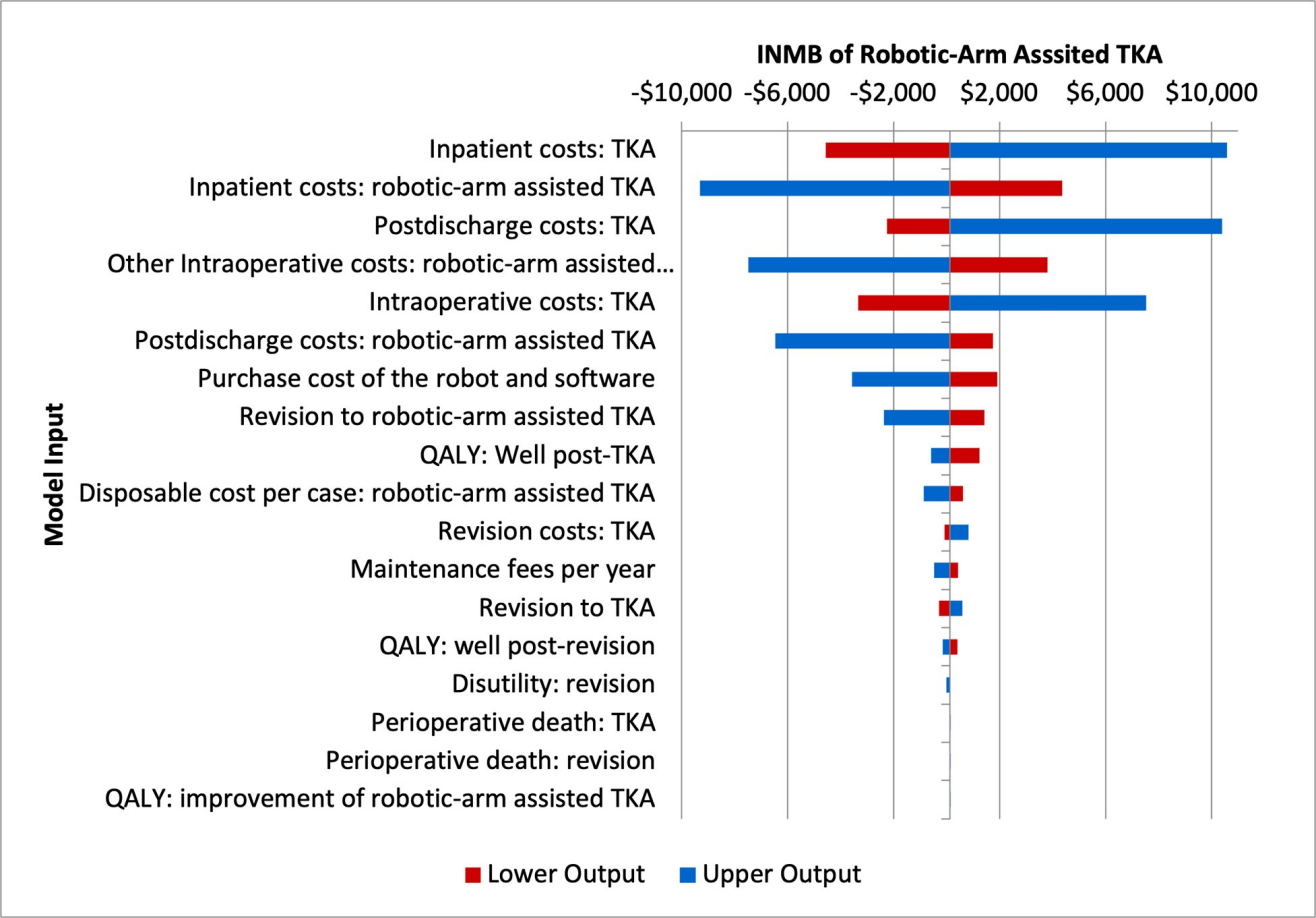
Important model parameters. **Abbreviations**: INMB: incremental monetary net benefit; TKA: total knee arthroplasty. **Note**: INMB tornado diagram for multiple 1-way sensitivity analyses. The INMB willingness-to-pay (WTP) threshold was assumed to be $50,000 per QALY. INMB was calculated as incremental QALYs × WTP−incremental costs. The importance of each model input on the economic conclusion is presented from top to bottom. The tails of each bar indicate the maximum and minimum INMB for each variable. A positive INMB indicates that the robotic-arm assisted TKA is cost-effective compared with conventional TKA at the given willingness-to-pay threshold.

Deterministic sensitivity analysis revealed variables that exerted a significant effect on INMB of robotic-arm assisted TKA (Fig 3). Overall, the cost-effectiveness of robotic-arm assisted TKA was most sensitive to cost measures of the primary procedure, including inpatient, intraoperative, and postdischarge costs. Robotic-arm assisted TKA was cost-effective if (1) inpatient costs of TKA were higher than $17,377; (2) inpatients costs of Robotic-arm assisted TKA were less than $16,066; (3) postdischarge costs of TKA were higher than $1,747; (4) intraoperative costs of TKA were lower than $11,100; (5) postdischarge costs of robotic-arm assisted TKA were less than $1,747; (6) purchase costs of the robot and software were less than $1,412,597 (7) revision rate of robotic-arm assisted TKA was lower than 0.46%; (8) QALY following primary TKA were less than 0.835 (9) Disposal costs of robotic-arm assisted TKA per case were less than $859; (10) costs of revision surgery were higher than $14,828. Notably, the cost-effectiveness of robotic-arm assisted TKA wasn’t sensitive to the assumption on QALY improvement introduced by the robotic technology. In our base case scenario, QALY weights following the initial TKA were assumed to be the same regardless of the use of robot assistance. We varied the improvement of QALY weights from 0 to 10 percent but INMB at a WTP threshold of $50,000 only changed from $129.55/QALY to $130/QALY.

## Discussion

This study used a decision-analytic model to evaluate cost-effectiveness of robotic-arm assisted TKA. The results suggested that robotic-arm assisted TKA was likely to be cost-effective relative to TKA in the Medicare-aged population at a hospital that performed more than 49 procedures per year. Though robotic-arm assisted technology requires a significant amount in fixed-asset investment (i.e., robotic system), high purchase costs can be offset by savings through reduced revision rates and fewer post-acute care costs.

Our sensitivity analysis revealed that high-volume hospitals may deliver higher value in performing in robotic-arm assisted TKA. Robotic-arm assisted technology requires a high initial investment in the equipment, high-volume hospitals can achieve economies of scale by spreading the investment over more TKA cases. Notably, deterministic sensitivity analysis demonstrated that costs that occurred in preoperative, intraoperative, and postoperative stages affected the results in wide ranges. In our model, transitional probabilities and costs were not modeled as a function of hospital volume. High-volume hospitals and surgeons were associated with improved patient outcomes and lower Medicare inpatient payments [33–38]. Therefore, high-volume hospitals have the potential of delivering robotic-arm assisted TKA more cost-effectively than our base case results suggested.

Though there is limited evidence on comparative effectiveness between robotic-arm assisted and conventional TKA, our study sheds light on key drivers of the incremental effectiveness of robotic technology. The one-way deterministic sensitivity analysis demonstrated that the robotic system needs to achieve lower than a 0.46% failure rate to be cost-effective. Early clinical evidence suggested that robotic technology has the potential benefits of delivering more accurate and precise bone cuts and implant positioning and offering soft tissue protection [11, 13, 16]. To more precisely measure the cost effectiveness of robotic-arm assisted TKA, more studies are needed to determine whether the technology will result in improved implant survivorship and long-term functional outcomes.

The study has several limitations. First, there might be hidden costs that we didn’t incorporate in the model regarding robotic-arm assisted technology. The surgical team, including the surgeon and the technical engineer, needs additional training to operate on the robotic arm and install software applications. Also, the surgical team has a learning curve to efficiently perform robotic-arm assisted TKA. The initial phase of the learning process might increase in the time of operation [39, 40]. Additional costs occur in both the training and extended operating time but there isn’t readily available data on the related costs. Second, we made a conservative assumption on the long-term outcomes following robotic-arm assisted TKA in the base case where QALY weights were assumed to be the same regardless the use of the robotic-arm technology. Under this assumption, the cost-effectiveness of robotic-arm assisted TKA might be underestimated. Third, we used cost estimates of the robotic system from a high-volume hospital and assumed that they are the same across hospitals. However, higher-volume hospitals may have more bargaining power and purchase the system at a lower price. Their cost-effectiveness to perform robotic-arm assisted TKA may be overestimated for lower-volume hospitals in our study.

Our model suggested that, under conservative assumptions on cost savings and QALY improvement, robotic-arm assisted TKA may be a cost-effective procedure in the Medicare aged population at high-volume hospitals with >49 procedures per year. The threshold identified may assist hospital administrators with deciding whether to adopt robotic technology in orthopedic surgery.

## Supporting information

S1 Data (XLSX)
